# Essential Oils in Skin Tissue Engineering: Opportunities, Integration, and Overcoming Challenges

**DOI:** 10.1007/s13770-026-00814-4

**Published:** 2026-07-17

**Authors:** Elif Emekdar, Selcen Ari Yuka, Azime Erarslan

**Affiliations:** 1 Faculty of Chemical and Metallurgical Engineering, Bioengineering Department, Yildiz Technical University, Esenler, 34210 Istanbul, Turkey; 2 Health Biotechnology Joint Research and Application Center of Excellence, Yildiz Technical University, Istanbul, Turkey

**Keywords:** Essential oils, Skin tissue engineering, Bioactive agents, Wound healing, Tissue regeneration

## Abstract

**Background:**

Essential oils (EOs) derived from plants have been utilized for the development of various approaches in biomedical applications due to their superior properties. In particular, their antimicrobial, anti-inflammatory, antioxidant, and wound-healing effects, alongside their biocompatibility and biodegradability, have made EOs a significant complementary agent in tissue regeneration-focused applications. Initial uses of EOs focused primarily on direct application and evaluation of their therapeutic efficacy; however, these approaches have been limited by issues such as volatility, instability, and potential for irritation.

**Methods:**

This review focuses on current perspectives in tissue engineering strategies, based on the biological functions of EO, such as biocompatibility, antimicrobial, anticancer, and antioxidant properties. Based on this comprehensive background of biological functions, current studies addressing nanoparticle systems, smart delivery systems, and wound dressings and coatings have been analyzed to identify existing issues and strategies to tackle these challenges.

**Results:**

In skin tissue engineering applications, innovative strategies such as nanoparticle encapsulation, integration into smart delivery systems, and the development of wound dressings containing EO have been developed. These advanced approaches offer advantages such as improved EO stability, controlled release, and enhanced efficacy, while also enriching the biofunctionality of the platform. While advanced delivery systems improve the stability of EOs, long-term stability under physiological and clinical conditions remains challenging. However, compared to conventional methods, these advancements strengthen the potential of EOs to drive tissue repair processes in a more controlled and functional manner.

**Conclusion:**

The integration of EO-based tissue engineering with nanotechnology offers a promising approach to optimizing the biological effects of these systems and enhancing the success of their application. Although challenges such as safety, biomaterial interactions, and scalability persist, recent advancements are rapidly overcoming these obstacles. EO-based strategies hold significant potential for overcoming current limitations and advancing the field, particularly in skin tissue engineering.

## Introduction

Essential oils (EOs), defined as complex mixtures of volatile bioactive compounds derived from plants, are of considerable interest in skin tissue engineering due to their diverse therapeutic properties such as antimicrobial, anti-inflammatory, antioxidant and wound healing effects [1]. These naturally derived components offer a promising alternative or complement to conventional synthetic agents, potentially enhancing tissue regeneration and reducing infection risks in wound treatment [2]. Due to these superior properties, various approaches have been developed to utilize EOs in standalone or integrated into tissue engineering applications, and their potential in future studies has been revealed.

EOs’ biological properties are the key factor for integrating EOs into tissue engineering applications. EOs possess biocompatible and biodegradable properties that contribute to tissue repair by promoting cell proliferation and migration, while also providing a safe environment for healing [3]. Their antimicrobial and anticancer activities help reduce infection and support healthy cell growth [4–6]. This section examines the ability of EOs to enhance cell proliferation and migration, alongside their antimicrobial and anticancer effects.

Initial applications of EOs primarily focused on elucidating their direct wound healing potential. However, this approach often revealed significant limitations, including their volatility, instability, and the tendency of pure forms to cause irritation or allergic reactions [7]. Consequently, recent studies have evolved toward developing innovative strategies to integrate EOs into advanced tissue engineering platforms [8]. Approaches such as encapsulation within nanoparticles, incorporation into smart delivery systems, and development of EO-loaded wound dressings and coatings have emerged, demonstrating promising results in preserving EO stability, controlling their release, and enhancing both safety and efficacy [3, 9, 10]. These advanced methodologies not only improve the bioactivity and sustained release of essential oils but also facilitate their combination with biopolymers and other biomaterials, thereby opening new horizons for diverse tissue engineering applications.

While these advances are encouraging, they have also introduced new challenges in the application of EOs within tissue engineering. Issues related to stability, volatility, interactions with biomaterials, formulation complexity, scalability, and cost-effectiveness continue to pose significant barriers to clinical translation [11–14]. Therefore, comprehensive and multidisciplinary approaches are required to optimize EO-based tissue engineering applications, ensure consistent therapeutic outcomes, and enable large-scale, cost-effective production. This review addresses the current developments of essential oils in skin tissue engineering within this framework, discusses emerging opportunities, and provides a comprehensive perspective on the challenges and potential solutions for future researches.

## The Role of Essential Oils in Skin Tissue Engineering

### Carrier Biocompatibility and Biological Effects of Essential Oils on Cell Proliferation and Migration

The discovery that EOs can promote tissue regeneration by inducing cell proliferation and migration has led to approaches to integrate them into biomedical applications due to these superior properties [15]. For example, *Lavandula angustifolia* EO has been reported to have no toxicity on foreskin fibroblast (HU02) cell lines and cell viability was above 90% [3]]. Another report revealed that *Lavandula angustifolia* EO regulates oxidative stress, which is critical for healthy wound healing and proliferation [16]. In addition, it was emphasized that Rosemary EO induced proliferation in HEK293 cells and therefore has potential for use in wound healing [17]. In a study conducted by Torabiardekani et al., the EO of *Zataria multiflora* was examined for its wound-healing effects and was reported to exhibit 95–100% cell viability on the NIH/3T3 cell line [18]. Similarly, a study by Osanloo et al. demonstrated that this EO was non-toxic to the L929 cell line and promoted reepithelialization and healing [19]. In a study by Dehkordi et al., *Tanacetum polycephalum* EO was evaluated for its wound-healing properties. The findings indicated that the oil was compatible with platelets, as the hemolysis rate was only 5%. Additionally, it was observed that the oil enhanced L929 fibroblast cell migration, reaching 100% in the fibroblast cell line [20]. In addition, EOs extracted from *Hypercium perforatum*, Coriender, *Bursera morelensis*, watermelon, jackfruit, and papaya have been reported to support cell viability and proliferation in fibroblasts and thus may contribute to wound healing [11, 21–23]. However, as the skin is a rich tissue composed of complex layers, well-designed studies addressing molecular processes in deeper detail are needed to understand the tissue healing mechanisms of EOs.

Due to the well-organized and complex structure of tissues, focusing solely on cell viability offers insufficient insight; therefore, various mechanisms, especially immune regulation, should also be investigated in assessing the potential of EOs in tissue healing. When *Cinnamomum burmanii* EO was tested on skin wounds by Zhang et al., 81.72% wound healing was observed at the highest concentration. It was reported that the highest proliferation was 149% in the human adult Keratinocytes, spontaneously immortalized (HaCaT) cell line and 134% in L929 cells. In the immune regulation aspect, it reduces interleukin-6 (IL-6), Tumor Necrosis Factor-alpha (TNF-α) secretion and M1 macrophage polarization (inflammatory phenotype) and increases M2 polarization (repair phenotype) in macrophages [24]. Alsakhawy et al. found that thyme EO promoted wound healing by increasing wound contraction, collagen accumulation, and re-epithelialization. The oil reduced IL-6 levels while boosting Transforming Growth Factor-beta 1 (TGF-β1) and Vascular Endothelial Growth Factor (VEGF) levels, enhancing angiogenesis and vascular permeability, ultimately facilitating wound healing [25]. Khezri's study on *Mentha pulegium* EO showed that it accelerated the proliferative phase of wound healing. The oil promoted cellular proliferation by increasing the expression of IL-10, Nuclear Factor kappa-light-chain-enhancer of activated B cells (NF-κB), TGF-β, and basic Fibroblast Growth Factor (b-FGF), leading to improved wound healing and a reduction in the inflammatory phase [26]. In a study on diabetic wound healing, it was stated that the Qiai EO reduced the levels of proinflammatory cytokine IL-6, contributed to angiogenesis by reducing the formation of reactive oxygen species (ROS) and accelerated healing [27].

The activity of EOs can be exhibited as concentration and content dependent manner. For example, while Lemongrass EO showed 80% cell viability when used at low concentration, it was reported that this viability decreased to 35% when used at high concentration and showed cytotoxic effect [13]. In a report that oregano EO achieved 90–96% cell viability in the L929 cell line, it has been reported that γ-terpinene, carvacrol, β-fenchyl alcohol and thymol components in the oil reduce Reactive oxygen species (ROS) levels, protect the cell from oxidative stress and contribute to wound healing [28]. The cinnamon leaf, tea tree and clove EOs were tested on keratinocytes and fibroblasts for wound healing, it was stated that cinnamon leaf EO and clove EO had little cytotoxic effect and increased cell metabolic activities in 48h. It was emphasized that cinnamon leaf oil may show toxic effects in fibroblasts due to its components such as eugenol and cinnamaldehyde, but the cells recovered from these effects after 48h. It was stated that tea tree EO did not show cytotoxic effect at low concentrations, but at high concentrations it caused IL-8 suppression in cells such as fibroblasts and in this case, cell compatibility with polymeric carriers was increased. It was also noted that both oils had strong antioxidant effects due to the eugenol content in clove and cinnamon EOs, with cinnamon leaf EO being higher, while tea tree EO had weaker antioxidant effects [29]. All this emphasizes the requirement for comprehensive analysis of the content of EOs due to their potential to modulate distinct molecular processes in tissue.

EOs exhibit content- and concentration-dependent biological activity and can influence the properties of platforms for tissue engineering. For example, tea tree oil enhanced phagocytic cell interaction and accelerated biomaterial reabsorption, i.e. increased the rate of biomaterial degradation, with most material absorbed within 90 days, though some lymphocytic inflammation and residue were noted. Despite this, no scarring or tissue disruption occurred during implantation, and the tissue healed without damage [30]. Developing approaches that induce EO-mediated cell proliferation and migration requires careful optimization, considering platform biocompatibility and biodegradability. However, EO-induced cell proliferation can have adverse effects, such as unintentionally promoting cancer cell growth. Therefore, understanding the molecular mechanisms of EOs is crucial for their safe use.

Collectively, the results indicates that EO-loaded delivery systems enhance fibroblast proliferation and migration primarily through modulation of oxidative stress and inflammatory signaling (Table 1). By reducing intracellular ROS levels and attenuating pro-inflammatory mediators, essential oils form a microenvironment conducive to cell survival and tissue repair. More importantly, these biological effects are closely linked to the physicochemical properties of the carrier system. Biocompatibility supports cell adhesion and viability, while biodegradability and controlled release govern the duration and consistency of EO exposure. With this, the regenerative efficacy of EO-based systems depends not only on the intrinsic bioactivity of the oils but also on the rational design of compatible and well-engineered carrier materials.

**Table 1:** Summary of essential oils and their effects on cellular responses and wound healing-related mechanisms

Origin	Subject	Key findings	Ref
Lavender (*L. angustifolia*)	HU02	No cytotoxicity (cell viability > 90%). Regulates oxidative stress and supports healthy wound healing	[3]
Rosemary	HEK293	Stimulates cell proliferation, accelerates wound healing	[17]
*Zataria multiflora*	NIH/3T3 and L929	No cytotoxicity (cell viability > 95%). Promotes re-epithelialization and tissue repair	[18, 19]
*Tanacetum polycephalum*	L929	Low hemolysis (~ 5%). Enhances fibroblast migration up to 100%	[20]
*Cinnamomum burmanii*	HaCaT and L929	Increases proliferation in both HaCaT (149%) and L929 (134%). Reduces inflammation via macrophage polarization from M1 to M2 phenotype	[24]
Thyme	Wound model	Enhances collagen deposition and increases VEGF regulation, while reducing IL-6 expression	[25]
*M. pulegium*	Wound model	Accelerates the proliferative phase of wound healing and increases IL-10 and TGF-β expression	[26]
Qiai EO	Diabetic wound model	Promotes angiogenesis by reducing pro-inflammatory cytokines and ROS generation	[27]
Lemongrass	General	Good viability profile at low concentrations (~ 80% viability)	[13]
Oregano	L929	No cytotoxicity (90–96% cell viability). Reduces oxidative stress	[28]
Cinnamon leaf & Clove	Keratinocytes and fibroblasts	Enriched antioxidant activity and favorable viability profile	[29]
Tea Tree	Keratinocytes and fibroblasts	Safe at low concentrations; suppresses IL-8 at higher concentrations and enhances phagocytic activity	[29]

### Antimicrobial Properties

In skin tissue engineering, preventing infection is a significant challenge due to the skin’s vulnerability to pathogens after injury. EOs are emerging as natural agents that can prevent infection, promote tissue regeneration, and exhibit antimicrobial properties. These oils are effective against various bacteria, including *Staphylococcus aureus (S. Aureus), Escherichia coli (E. Coli)*, and *Pseudomonas aeruginosa (P. aeruginosa)*, as well as fungi like *Candida albicans (C. Albicans)* [31]. Most of them mediate this effect by disrupting cell membranes, inhibiting protein synthesis, and disrupting metabolic processes. EOs such as tea tree, lavender, and cajeput have been shown to improve wound healing by reducing infection risk and promoting tissue repair [4, 31]. Their integration into biomaterials can enhance wound healing outcomes by preventing infections and supporting tissue regeneration [32].

Several studies have investigated the antimicrobial effects of EOs on wound healing and infection (Table 2). Dehkordi et al. reported that *Tanacetum polycephalus* EO effectively killed both of *S. aureus* and *E. coli* by disrupting bacterial membrane permeability and inactivating bacterial enzymes [20]. Lavender oil demonstrated strong antimicrobial activity via damage the cell membrane against *S. aureus* and *C. albicans*, while its effect on *E. coli* was less pronounced [3, 16]. Fawal et al. investigated the use of oregano EO as a wound dressing and found an inhibition rate of 82.3% against *S.* aureus and 67% against *E. coli.* These results highlighted the strongest effect on *S. aureus*. The antimicrobial action of oregano EO was attributed to the penetration of its bioactive components into the cell's phospholipid bilayer, which disrupted cell metabolism and led to cell death [28]. EOs with antibacterial properties are not limited to these; tests of *Cinnamomum burmanni, Momordica charantia, Hypericum perforatum,* rosemary, Qiai EOs on *S. aureus* and *E. coli* have also shown that they can be used for their superior antibacterial properties in wound healing [17, 21, 24, 27].

**Table 2:** Essential oils, antimicrobial effects and strategies to enhance efficacy with advanced applications

Essential oil	Study design
*Tanacetum polisefali*	**Strain:*** Staphylococcus aureus* (ATCC 25923),* Escherichia coli* (ATCC 25922)** Extraction method:** Hydrodistillation ** Antimicrobial mechanism:** disruption of membrane permeability and protein saturation reduction, causing cell death ** Rate:** 99.99% of both gram-negative and gram-positive bacteria in disk diffusion test [20]
*Lavanda angustifolia*	**Strain:*** Staphylococcus aureus, Candida albicans, Escherichia coli* **Extraction method:** Hydrodistillation **Antimicrobial mechanism:** Damaging the cell membrane **Rate:** Strong antimicrobial effect against *S. Aureus* (18.90 ± 1.69) and *C. Albicans (*20.25 ± 2.47), weak effect against *E. Coli (*15.10 ± 1.55) in disk diffusion [16]
*Lavandula angustifolia*	**Strain:*** Staphylococcus aureus,* **Extraction method:** Not specified **Antimicrobial mechanism:** Not evaluated **Rate:** MIC values of Lav-O, Lav-SLN and Lav-SLN-G were 0.12 mg/ml, 0.05 mg/ml and 0.045 mg/ml in microdilution analysis [3]
Lemongrass	**Strain:** *Candida albicans, Staphylococcus aureus, Escherichia coli* **Extraction method:** Commercial **Antimicrobial mechanism:** Reduced the expression of the agrA and α-toxin encoding (hla) genes and suppressed the genes responsible for fatty acid and peptidoglycan biosynthesis **Rate:** Over 99% effectiveness in minimum inhibition concentration [13]
Oregano	**Strain:*** Staphylococcus aureus, Escherichia coli* **Extraction method:** Commercial **Antimicrobial mechanism:** Affecting cell metabolism and leading to cell death **Rate:** 82.3% inhibition against *S. aureus* and 67% inhibition against *E. coli* in disk diffusion. [28]
*Cinnamomum burmanni*	**Strain:** *Escherichia coli* (ATCC 25922), *Staphylococcus aureus* (ATCC 25923) **Extraction method:** Commercial **Antimicrobial mechanism:** Not evaluated **Rate:** The minimum inhibitory concentration (MIC) and minimum bactericidal concentration (MBC) were determined [24]
*Momordica charantia, Hypericum perforatum*	**Strain:*** Escherichia coli, Staphylococcus aureus* **Extraction method:** Commercial **Antimicrobial mechanism:** Bacterial membrane damage, increased cell permeability, coagulation of bacterial proteins, and inhibition of ATP production **Rate:** Average inhibition zones of 14.05 mm and 12.70 mm for *E. coli* and *S. aureus*, respectively in disk diffusion method [21]
Rosemary	**Strain:** *Escherichia coli* (DH5α), *Staphylococcus aureus* (ATCC 29213) **Extraction method:** Commercial **Antimicrobial mechanism:** Attachment to the microbes cell membrane and subsequently penetrates the respiratory chain, leading to the complete shutdown of oxidative phosphorylation **Rate:** 13.83 ± 0.05 mm for *E. Coli* and 9.96 ± 0.11 mm for *S. aureus* [17]
Qiai	**Strain:** *Escherichia coli* (ATCC 25922), *Staphylococcus aureus* (ATCC 29213) **Extraction method:** Distillation **Antimicrobial mechanism:** Disruption of the bacterial membrane, causing leakage of cytoplasm and proteins **Rate:** 70.5% against *E. coli* strains and 80.6% against *S. Aureus*. When NIR was applied, the antimicrobial effect increased to 96.6% for *E. coli* and 98.6% for *S. aureus*. [27]
*Salvia officinalis L. (sage), Rosmarinus officinalis L.* (rosemary), *Commiphora myrrha Nees Engl.* (myrrh), *Origanum majorana L.* (marjoram), *Pelargonium zonale L. L’Hér. ex Aiton* (geranium), *Chrysanthemum morifolium Ramat.* (chrysanthemum)	**Strain:** *Cutibacterium acnes (C. acnes)* (ATCC 6919) **Extraction method:** Hydrodistillation **Antimicrobial mechanism:** Not evaluated **Rate:** Marjoram and chrysanthemum EOs MIC < 0.2% v/v. Sage, rosemary, and myrrh EOs MIC > 0.2% v/v, Geranium EO no antibacterial effect [33]
*Lavandula angustifolia, Mentha piperita*	**Strain:** *Cutibacterium acnes* CCM 3437, *Staphylococcus epidermidis (S. Epidermidis)* CCM 4418 **Extraction method:** Commercial **Antimicrobial mechanism:** Destabilizes the permeability of cell membranes, diminishes the ergosterol rates, and consequent reduction throughout the production of PM-ATPase **Rate:** Lavender S Provence showed favourable antimicrobial efficiency against *S. Epidermidis (*6.3 ± 1.0 µl/ml) *and C. Acnes (*6.3 ± 1.0 µl/ml) [34]
*Satureja mutica, Oliveria decumbens*	**Strain:** *Escherichia coli* (ATCC 25922), *Staphylococcus aureus* (ATCC 25923), *Pse*udomonas aeruginosa (ATCC 9027), *Candida dubliniensis (C.dubliniensis)* (CBS 8501), *Candida albicans* (ATCC 10261) **Extraction method:** Hydrodistillation **Antimicrobial mechanism:** Debilitating of the cell membrane **Rate:** The monoterpene components such as carvacrol and thymol inhibited the growth of *E. coli*, *S. aureus*, and *P. aeruginosa* bacteria and exhibited antifungal activity against *Candida dubliniensis* (CBS 8501) and *Candida albicans* (ATCC 10261). The highest efficiency was observed against *Candida dubliniensis* with Satureja mutica EO at 0.0625 µl/ml and Oliveria decumbens at 0.125 µl/ml [35]
*Zataria multiflora, Cuminum cyminum*	**Strain:*** Escherichia coli, Pseudomonas aeruginosa, Staphylococcus aureus* **Extraction method:** Commercial **Antimicrobial mechanism:** Disrupt the bacterial membrane, inhibit efflux pumps, and prevent protein synthesis **Rate:** When tested against *E. Coli* 178 μg/mL, *P. Aeruginosa* 95 μg/mL, and *S. Aureus* 307 μg/mL, *Z. multiflora* EO showed stronger antimicrobial activity compared to *C. cyminum* EO [5]
*Zataria multiflora*	**Strain:** *Candida albicans* (CBS562), *Candida dubliniensis* (*C. Dubliniensis*) (CBS 8501), *Candida glabrata* (*C. glabrata*) (ATCC 90030), *Candida tropicalis* (*C. tropicalis*) (ATCC 750), *Candida parapsilosis* (*C.parapsilosis*) (ATCC 4344), *Candida krusei (C. krusei)* (ATCC 6258) **Extraction method:** Commercial **Antimicrobial mechanism:** Disrupting fungal cell wall or membrane integrity and interfering with ergosterol biosynthesis **Rate:** While ZM EO produced significant fungistatic activity against *C. Dubliniensis* and *C. Parapsilosis*, their integration into PVA/CS/Gel hydrogel increased the fungistatic activity of the platforms up to 16-fold against all strains [18]
*Mentha pulegium*	**Strain:** *Staphylococcus epidermidis, Salmonella Typhimurium (S. Typhimurium), Staphylococcus aureus, Listeria monocytogenes (L. monocytogenes), Escherichia coli, Pseudomonas aeruginosa* **Extraction method:** Hydrodistillation **Antimicrobial mechanism:** The lipophilic ends of lipoteichoic acids in these bacteria facilitated the penetration of hydrophobic compounds, allowing interaction with EOs and resulting in antimicrobial activity **Rate:** Not reported Antibacterial activity was reported against *S. epidermidis*, *S. aureus*, *L. monocytogenes* (gram-positive bacteria), and *E. coli* and *P. aeruginosa* (gram-negative bacteria). The highest MIC and MBC values were observed against *E. coli*, *P. aeruginosa*, and *S. Typhimurium.* When integrated with a nano lipid carrier, the MIC for *S. Typhimurium* showed a two-fold decrease [26]
Seeds of watermelon, jackfruit, and papaya	**Strain:** *Methicillin-resistant Staphylococcus aureus, Pseudomonas aeruginosa* **Extraction method:** Ultrasonic extraction **Antimicrobial mechanism:** Not evaluated **Rate:** Significantly stronger antibacterial activity against *Methicillin-resistant Staphylococcus aureus* and *P. aeruginosa* was observed for all extracts. [23]
*Thymus vulgaris*	**Strain:** *Staphylococcus aureus, Sta*phyloc*occus epidermidis, Escherichia coli* **Extraction method:** Hydrodistillation. Antimicrobial mechanism: Increased permeability in the bacterial cell membrane, leading to alkaline phosphatase leakage and membrane damage. Nucleic acid and protein leakage was higher in gram-negative bacteria. The antimicrobial effect was attributed to cell wall and cytoplasmic membrane disruption rather than targeting bacterial DNA **Rate:** MIC values of 0.375 mg/mL for *S. aureus*, *S. epidermidis*, and *E. coli*, and 0.75 mg/mL for *P. aeruginosa*. [25]
*Mentha longifolia, Mentha pulegium, Zataria multiflora*	**Strain:*** Staphylococcus aureus, Pseudomanas aureginosa* **Extraction method:** Commercial **Antimicrobial mechanism:** Not evaluated **Rate:** Over 90% in *S. aureus* and over 60% in *P. Aeruginosa* [36]
Oregano, Euganol	**Strain:** *Staphylococcus aureus* (ATCC 25923), *Listeria monocytogenes* (CMCC 54004) **Extraction method:** Not specified **Antimicrobial mechanism:** Disrupts the bacterial cell membrane, alters its permeability, alters the dynamic flow of protons within the cell **Rate:** Eugenol exhibits comparable efficacy against *S. aureus* and *L. monocytogenes*, while oregano is more effective against *S. aureus*. The combined use of two EOs significantly increases the efficacy [10]

Since the habitat of microorganisms varies according to the location of the treatment, the bacterial diversity for analyzing the effectiveness of EOs can be addressed more extensively in current studies (Table 2). Khezri et al. reported that *Mentha pulegium* EO exhibited antibacterial activity against gram-positive bacteria of *S.epidermidis, S. aureus* and *L. monocytogenes* and negative bacteria of *E. coli* and *P. aeruginosa*, with gram-positive bacteria being more sensitive. The oil's antimicrobial effect was attributed to its interaction with bacterial lipophilic structures, facilitating penetration [26]. For example, *C. acnes* and *S. epidermidis* were used to evaluate the antibacterial properties of various EOs for skin applications and it was found that antibacterial activity can be achieved in a concentration-dependent manner for the inhibition of these specific strains [33, 34]. However, this rich population is not only limited to bacterial strains, but fungal agents should also be considered in these studies. In a study by Torebiardekani et al., it was observed that *Zataria multiflora (Z. multiflora)* EO showed antifungal activity against 6 standard yeast strains including *C. albicans* (CBS562), *C. dubliniensis* (CBS 8501), *C. glabrata* (ATCC 90030), *C. tropicalis* (ATCC 750), *C. parapsilosis* (ATCC 4344) and *C. krusei* (ATCC 6258). It was stated that this effect was thought to be due to the thymol in the oil that may disrupt the integrity of the fungal cell wall or membrane and interfere with ergosterol biosynthesis [18].

The most remarkable aspect of antimicrobial efficacy studies of EOs is that the efficacy is generally concluded to be achieved by disruption of the cell membrane [23, 25]. However, alternative mechanisms of bacterial death should also be considered. For example *Zataria multiflora* EO showed stronger antimicrobial activity than *C. cyminum(Cuminum cyminum)* against *E. coli*, *P. aeruginosa,* and *S. aureus*, likely due to its carvacrol and thymol content, which disrupts bacterial membranes, inhibits efflux pumps, and prevents protein synthesis[5]. When lemongrass EO was examined, it was stated that it showed an antimicrobial effect of over 99.60% against *C. albicans, S. aureus* and *E. coli*. This effect was observed that the citral component in lemongrass EO reduced the expression of accessory gene regulator A (agrA) and α-toxin coding (hla) genes. It was also emphasized that it suppressed the genes responsible for fatty acid and peptidoglycan biosynthesis and weakened the survival mechanism of the bacteria [13]. To advance skin tissue engineering applications with enhanced antimicrobial properties, comprehensive studies are needed that systematically evaluate the composition of EOs in relation to microbial diversity across a broad target range. Additionally, these studies should be supported by robust experimental approaches to elucidate the efficacy of EOs in bacterial cell death processes.

### Anticancer Properties

**Fig. 1:**
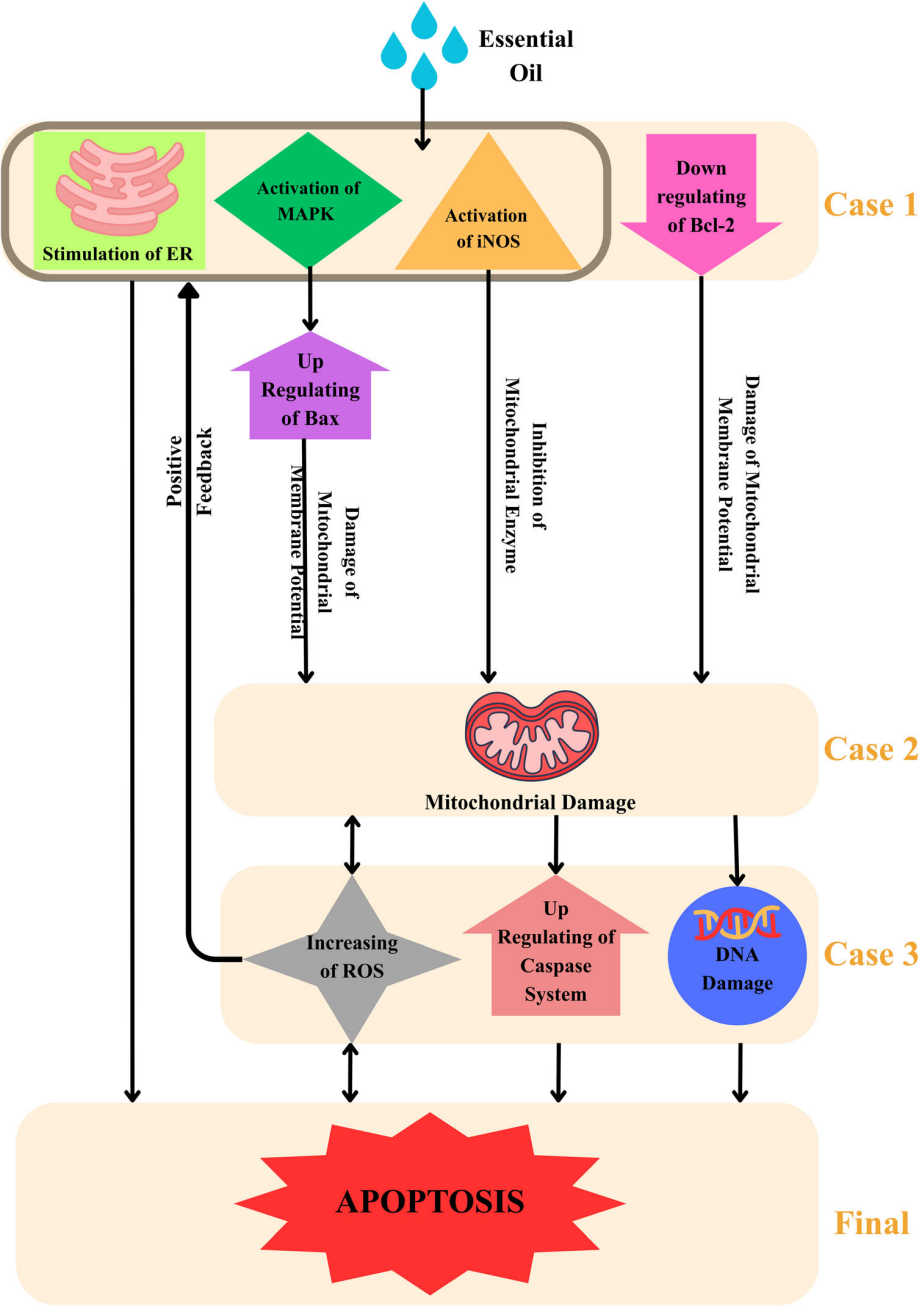
Proposed anticancer mechanisms of EOs after cellular internalization. Following cellular entry, EOs activate multiple intracellular signaling pathways that collectively lead to apoptosis. These include stimulation of endoplasmic reticulum (ER) stress, activation of MAPK signaling, and induction of inducible nitric oxide synthase (iNOS). These events result in upregulation of pro-apoptotic Bax and downregulation of anti-apoptotic Bcl-2, leading to mitochondrial membrane potential disruption. Subsequent mitochondrial damage promotes ROS generation, activation of the caspase cascade, and DNA damage, ultimately triggering apoptotic cell death

Skin cancer is one of the most prevalent malignancies worldwide. Bioactive compounds found in EOs hold significant potential to enhance existing treatment strategies and pave the way for novel therapeutic approaches due to their antioxidant, antimicrobial, and anticancer properties. Increasing evidence suggests that EOs play a pivotal role in tumor suppressor mechanisms, promoting healthy cell proliferation and offering promising avenues for cancer therapy. Figure 1 schematically illustrates the proposed anticancer mechanisms of essential oils at the cellular level. Following cellular internalization, EOs may induce endoplasmic reticulum stress, activate MAPK signaling pathways, and disrupt mitochondrial membrane potential, leading to enhanced ROS generation, caspase activation, and DNA damage. These interconnected processes ultimately trigger apoptosis, providing a mechanistic basis for the anticancer potential of EOs. Supporting this mechanistic framework, *Rosmarinus officinalis* L. EO and α-pinene (identified major compound) reduced the viability of melanoma skin cancer cells by more than 90%. It was emphasized that this was accomplished through the regulation of Bax (pro-apoptotic gene) and Bcl-2 (anti-apoptotic gene) expression [37]. In another report examining the efficacy of neomenthol on skin cancer, it was found to inhibit the proliferation of human epidermoid carcinoma (A431) cells and increase sub-diploid cells by arresting the G2/M phase in skin cancer. It has also been reported to inhibit biomarkers of progression, promotion, and initiation stages of the carcinogenesis process, disrupt mitochondrial membrane potential (MMP) and induce apoptosis by increasing ROS. Moreover, In the Ehrlich ascites carcinoma (EAC) model, it was observed that it was safe up to 1000 mg/kg of body weight, 58.84% at 75 mg/kg and 23.98% at 50 mg/kg bw i.p. dose EAC tumor cells were reduced [38]. In another study, *Z. multiflora* EO was observed to have a higher cytotoxic effect on A375 and A431 cancer cells compared to *C. cyminum*. The IC50 value of *Z. multiflora* EO for A-375 cells was 132 μg/mL and for A-431 cells was 158 μg/mL, while the IC50 value of *C. cyminum* EO for A-375 cells was 664 μg/mL and for A-431 cells was 321 μg/mL [5]

Remarkably, while there are very rare reports of EOs in skin cancer applications, there have been significant findings in other cancer types. Evaluation of the activity of carvacrol in the EOs of *Origanum majorana, Origanum vulgare, Thymus vulgaris* and *Lippia graveolens* on several cancer cell lines (FaDu, K562 and A549) showed IC50 values of 9.61 ± 0.05 and 81.32 ± 11.83 μM, respectively [39]. In another recent study, it was stated that the cell cycle was arrested in the G2/M phase and the anticancer effect emerged by increasing the expression of apoptosis-related genes, the thymus vulgaris oil was treated on human hepatocellular carcinoma cell line (HepG2). It has been determined that EO triggers apoptosis (programmed cell death) by activating oxidative stress and ER stress pathways and exhibits a potent anticancer effect in a dose-dependent manner. [40]. The active components of EOs have not yet been fully identified, and their integration into modern tissue engineering applications remains limited, particularly in areas such as skin cancer. [41]. However, considering the common dysregulated mechanisms in cancer types, these new findings in other cancers may open new horizons for skin cancer as well.

### Antioxidant Properties

Oxidative stress plays a significant role in wound healing and skin tissue degeneration. Excessive accumulation of ROS in the wound area disrupts cellular homeostasis, damaging lipids, proteins, and nucleic acids. It inhibits fibroblast proliferation, keratinocyte migration, and extracellular matrix remodeling. Controlled ROS production is essential for antimicrobial defense and signaling in the early stages of healing, but persistent oxidative imbalance leads to chronic inflammation and delayed tissue repair. In this context, essential oils are promising due to their ability to largely scavenge free radicals and modulate redox pathways. The incorporation of EOs into biomaterial-based scaffolds and advanced delivery systems provides structural support and also facilitates the biochemical regulation of the wound microenvironment.

For example, PCL/MEO structures developed by incorporating essential oil from Melissa officinalis into poly(ε-caprolactone) (PCL) membranes demonstrated radical scavenging activity with approximately 24-45% concentration-dependent inhibition in DPPH assays. The preservation of antioxidant activity after integration into the polymeric matrix indicates that functional stability has been achieved. It was observed that moderate essential oil concentrations maintained viability in L929 fibroblast cells above 87%, and cellular activity decreased at higher loadings. This highlights the importance of dose optimization [42]. In another study, essential oil obtained from *Zataria multiflora* was integrated into a bilayer electro-spun structure containing type I collagen (inner layer) and collagen/PLLA (outer layer). The essential oil showed strong free radical scavenging activity with an IC_50_ value of 7.4 ppm in DPPH analyses. This high antioxidant effect was attributed to the redox regulatory effects of its main phenolic components, carvacrol and thymol, through hydrogen donation and ROS neutralization. Integration of the essential oil into the nanofiber scaffold ensured the preservation of antioxidant functionality and provided an initially rapid release followed by a controlled release profile for up to 72h. This release kinetics provides an advantage in the early wound phase where oxidative stress is most intense [43]. In a study where the structure was stabilized in chitosan oleate-based nanoemulsions obtained from *Syzygium aromaticum*, in vitro DPPH analyses showed that the fat had radical scavenging activity. Cellular-level redox modulation was confirmed in fibroblast cultures exposed to H_2_O_2_-induced oxidative stress using electron paramagnetic resonance (EPR) imaging and a spin-trap system, and a significant ROS signal was observed in fibroblasts exposed to oxidative damage. Intracellular radical formation was reported to be reduced. The fact that this effect can be detected before cytotoxicity occurs emphasizes its early-stage redox buffering capacity. In a murine burn model, the structure improved macroscopic wound closure and enhanced histological repair after 18 days. In addition, TBARS analysis showed a decrease in lipid peroxidation levels and suppression of *in vivo* oxidative damage [44]. Berechet et al. determined that nanofibers produced by electrospinning from hydrolyzed bovine collagen and loaded with ginger essential oil from *Zingiber officinale* had a DPPH radical scavenging capacity of approximately 79.8%. This antioxidant effect is largely attributed to the phenolic components of the essential oil. These compounds neutralize free radicals thanks to their hydrogen or electron-donating properties. Because collagen is a key structural component of the dermal extracellular matrix, its combination with the antioxidant essential oil offers a structure that mimics natural tissue architecture. Additionally, it forms a biofunctional scaffold that actively modulates oxidative stress. This dual functionality is considered to provide a significant advantage in skin tissue engineering applications where oxidative damage can inhibit matrix accumulation and cellular migration [45].

Current data suggest that the antioxidant activity of essential oils represents a critical but underemphasized mechanism in skin tissue engineering. The antioxidant effect works by scavenging excess ROS, reducing lipid peroxidation, and protecting fibroblasts from oxidative damage, thereby supporting a more balanced regenerative microenvironment. However, further detailed research is needed to clarify the optimal dose, long-term redox stability, and mechanistic pathways to ensure safe and reproducible clinical translation. Overall, antioxidant modulation is a significant biological function of essential oils, complementing properties such as antimicrobial and proliferative effects in advanced structures.

## How to be Entegrated Essential Oils into Advanced Tissue Engineering Approaches

EOs play crucial role in wound healing and skin tissue engineering studies, especially due to their biological effects. However, direct use of EOs creates various problems to the body. Due to their volatile and unstable structures, EOs quickly evaporate and lose their effects [7]. They cause allergic reactions or irritation in their pure forms. For these reasons, using pure form of EOs reduces the bioavailability of the oil. In order to overcome these challenges, EOs need to be used in different forms and with various systems. In this way, safer use is provided by preserving the stability of the oil and increasing its effectiveness. In this section, nanoparticle systems, smart delivery systems and wound dressing and coatings studies used to prevent these problems are mentioned.

### Nanoparticle Systems

Nanoparticle systems are used for the safe use of EOs in skin tissue engineering because higher efficiency is achieved by preserving the form of the EO used. They are also used to increase the effectiveness of wound dressings containing nanoparticles with EOs. The use of oil in nanoparticles to increase the efficiency of EOs is examined in this section, and studies on wound dressings containing EOs supported by nanoparticles are discussed under the section Wound Dressing and Coatings.

EOs may be chemically instable in the presence of air, light, moisture and temperature. In a study conducted by Fahimnia et al. to overcome instability issue, *Lavandula angustifolia* EO was loaded into solid lipid nanoparticles. It was observed that the small-sized particles increased the stability and bioavailability of EO. The inclusion of lavender oil in this lipid matrix provided a retention efficiency of 75.46% and released 75.40% in 72h, thus providing a controlled and long-term release, reducing the risk of skin irritation and providing a more effective treatment [3]. In another study, *Cuminum cyminum* and *Zataria multiflora* EOs were loaded into alginate nanoparticles to protect the volatility and degradation of EO. It was stated that nanoparticles showed higher activity compared to other nanoformulations [5]. Similarly, a study conducted by Rahmeni et al. has stated that the activity of *Rosmarinus officinalis* *L*. EO increased when integrated into chitosan NP system [37]. Saraiva’s study on gel formulation of chitosan nanoparticles with *Helichrysum italicum* EO showed that the penetration of the EO into the tissue is increased, continuous release is provided. It was also stated that it reduces the oxidation and photothermal degradation rate of the EO and observed that the structure of this formulation remained stable for 2–3 months under various storage conditions and the gel improved skin moisture and maintained healthy skin conditions at least 60 min after application to the skin. Thus, it was stated that it has potential in cosmetic applications and is a suitable treatment for inflammatory skin diseases [46]. Developing approaches that improve the stability and controlled release of EOs requires appropriate optimization because dosage form of EO should be sufficient and biocompatible to effect on skin. Also, deep understanding are needed about mechanisms of efficiency and should be supported by compherensive studies.

### Smart Delivery Systems

**Fig. 2:**
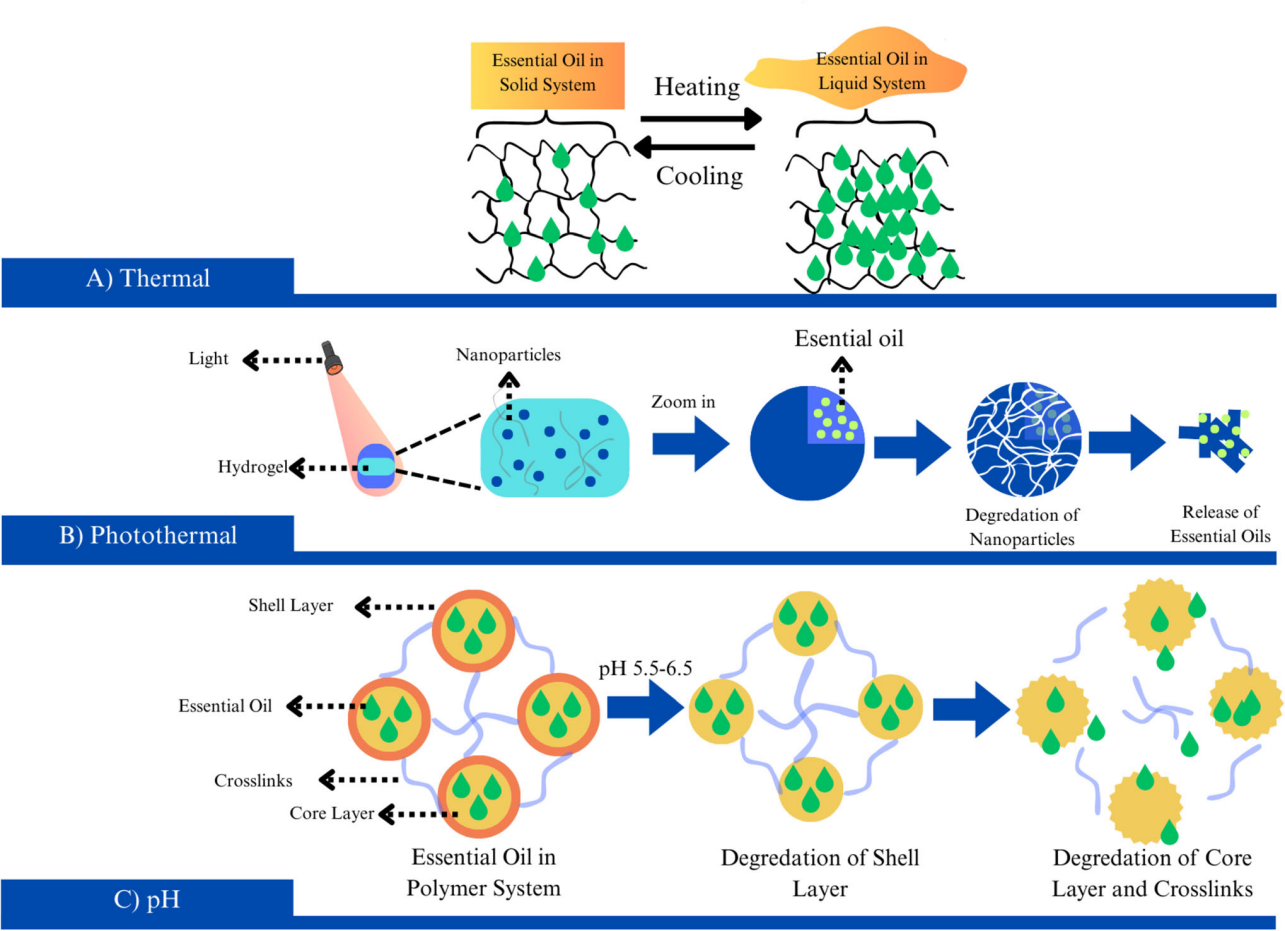
Smart delivery systems for stimuli-responsive release of essential oils in skin-related applications. **A** Thermoresponsive systems, in which temperature-induced phase transitions between solid and liquid states regulate the retention and release of essential oils. **B** Photothermal-responsive systems, where light irradiation activates embedded nanoparticles, generating localized heat that promotes nanoparticle degradation and controlled essential oil release. **C** pH-responsive polymeric systems, in which acidic microenvironments (pH 5.5–6.5) trigger degradation of the shell and core layers as well as crosslinks, enabling the release of encapsulated essential oils. These smart systems allow on-demand, site-specific delivery while improving essential oil stability and therapeutic efficiency

The system used to increase the effectiveness of EOs in skin tissue engineering is designed to provide effectiveness in the presence of stimuli such as temperature and pH (Fig. 2). This smart responsiveness ensures that the EOs are released and activated precisely according to the specific conditions of the application site, optimizing their therapeutic impact. By enabling controlled and targeted delivery, the system not only increases the bioavailability of the EOs but also minimizes potential side effects.

With these smart delivery systems, the chemical stability of EOs is preserved and sufficient effect is provided in the targeted area. For example, in a study conducted by Torabiardekani et al., *Zataria multiflora* EO which has toxic effect when applied directly to skin, was encapsulated in a polyvinyl alcohol/chitosan/gelatin hydrogel as a positive thermo-sensitive to increase the bioavailability, effect and protect the structure of EO. It was observed that these hydrogels, which have a phase transition temperature close to human body temperature, dissolve on contact with warm skin, providing a non-invasive application. It was showed that the hydrogels show a gel-sol viscoelastic transition at 24.5 °C, allowing facilitated release of the EO, thus exhibiting a sudden release to initially reduce the activity of pathogens [18]. Similarly, a thermal stimulus-sensitive gel was designed using high-level *Copaifera reticulata* oil-resin combinations. It was reported that it increased skin permeability due to the bioadhesive effect and formulation remained stable for two years. It was increased the effectiveness of the components with its thermo-stimulus-sensitive structure (Fig. 2A) [47].

The effectiveness of EOs is also increased by photothermal stimulation (Fig. 2B). For example, a hydrogel sensitive to photothermal stimulation was developed by encapsulating Qiai EO in graphene oxide, because Qiai EO is difficult to use due to its hydrophobicity, volatility and poor water solubility. It was observed that near-infrared (NIR) irradiation increased the release of EO in hydrogels. After a 5 min NIR irradiation, a significant increase in the release of EO was observed. After 3 irradiation cycles, the cumulative release of EO reached 43.85%, and it was observed that 92.11% of the oil was released at the end of 128h. It was concluded that the combination of EO and graphene oxide affected the properties of the oil more strongly when exposed to NIR laser. So, it can increase wound healing and antibacterial properties [27].

As one of the important specific parameters of tissues or conditions, pH is frequently used in the development of the most useful approaches to smart system design (Fig. 2C). Especially, it plays crucial regulatory role in wound healing. For this reason, the pH sensitivity of the system to be designed increases the effectiveness of the active substance by supporting wound healing. pH-sensitive carriers trigger the release of EOs in response to changes in the acidic or basic environment, allowing the oils to act specifically in infected or inflamed areas. This targeted release enhances bioavailability while minimizing the risk of side effects. For example, in a study conducted by Sahu et al., a drug carrier system was prepared for skin cancer. 5-Fluorouracil (5-FU) was encapsulated in PLGA, coated with cationic chitosan, and subsequently combined with eucalyptus EO. Thus, it was reported that it increased the interaction with the negatively charged skin cancer cell membrane and ensured that the nanogel could effectively pass through the skin barrier. It was stated that cationic chitosan interacted with the negatively charged lactic acid formed due to low pH in the tumor microenvironment and 5-FU was released in a controlled manner [48]. In a more recent study by Qiu et al. a hyaluronic acid and carboxymethyl chitosan hydrogel dressing containing eugenol and oregano EO that can respond to wound pH was developed. It was reported that it showed 4 times more degradation in an acidic environment than in a neutral environment due to its pH sensitivity. It was showed that the eugenol release increased from 37.6% to 82.1%. Thus, this system was observed to enhance the efficiency of the EO and accelerate wound healing, offering significant advantages, although the hydrogel must be replaced frequently due to its biodegradability [10].

Integration of EOs with smart carrier systems enables targeted and controlled drug delivery, providing significant advantages in applications such as wound healing, infection control and treatment of many diseases. These innovative approaches enable the potential of EOs to be used more efficiently and constitute a promising platform in the field of skin tissue engineering. However, the design and production of these smart systems may involve high costs and complex processes in terms of large-scale production. At the same time, the volatile and unstable structures of EOs may make it difficult to maintain their stability in the system. In addition, the effects of photothermal or pH-sensitive mechanisms on natural changes in the body environment may increase the risk of off-target release. The limited number of *in vivo* studies on the biocompatibility and toxicity profiles of these systems constitutes a significant obstacle to transition to clinical applications.

### Wound Dressings and Coatings

Since the direct application of EOs can lead to various adverse effects, wound dressings and coating materials have been developed to ensure their controlled and effective use. These systems play a critical role in the field of skin tissue engineering; wound dressings and coatings maximize the biological benefits of EOs at a localized site while optimizing their efficacy through a controlled release mechanism. They also support the healing process by protecting the wound site from environmental factors, increase the stability of EOs and ensure their safe use; thereby effectively and reliable evaluation of the therapeutic potential of EOs.

Since the unstable and brittle structure of EOs makes their use difficult, they might be encapsulated in hydrogel matrices to maintain their biological activity, increase their effectiveness, maintain their stability and provide controlled release (Table 3). For example, Vasile et al. developed a wound dressing by coating hydrogels with zinc oxide and EOs of thyme and clove to address the volatility and low stability of EOs. This dressing promoted rapid wound healing without infection risk, with the zinc oxide and EO coating enhancing its function and effectiveness. Notably, samples containing thyme oil exhibited a higher degradation rate compared to those with clove oil [58]. The wound dressings have been formed by cross-linking eucalyptus, ginger, and cumin EOs to hydrogels, with the eucalyptus-containing samples notably accelerating burn wound healing to approximately 48h through the effective use of these oils [54]. In the study by Hasempur et al., a creatine-gelatin cryogel containing *Zataria multiflora* EO and titanium dioxide nanoparticles promoted wound healing by releasing a significant amount of EO early to reduce infection, followed by sustained delivery, which decreased dressing change frequency and supported cellular integrity during tissue regeneration [49]. It has been reported that the use of sodium alginate hydrogel patch containing peppermint EO and zinc oxide nanoparticles can create a cooling effect as well as rapid wound healing [50].

**Table 3:** Formulation strategies and mechanistic insights of essential oils in tissue repair

Essential oil	Formulation and evaluation strategy	Mode of action in tissue repair
*Zataria multiflora*	A creatine-gelatin cryogel containing *Zataria multiflora* EO and titanium dioxide nanoparticles was produced	Initial burst release of EO reduced infection, followed by sustained delivery; enhanced tissue regeneration, cellular integrity, and functional recovery [49]
*Mentha piperita*	*Mentha piperita* was extracted by distillation. A sodium alginate hydrogel patch containing the obtained EO and zinc oxide nanoparticles was created	Patch application induced a cooling effect, accelerated wound closure, and improved healing via synergistic action with nanoparticles [50]
*Lavandula angustifolia*	Polyurethane nanofibers produced by the electrospinning method were simultaneously loaded with lavender oil and silver nanoparticles to create a wound dressing	Incorporation of the oil enhanced the hydrophobicity of the wound dressing, promoting moisture retention and facilitating wound healing [9]
Saqez	A hydrogel wound dressing containing *Saqez* oil, polyvinyl alcohol, and chitosan was produced	The oil reduced swelling and water vapor permeability of the hydrogel, increased surface water retention, and enhanced hydrophobicity; cell viability remained above 80%, supporting wound repair [51]
*Calophyllum inophyllum*	*Calophyllum inophyllum* loaded Poly (ε-caprolactone) (PCL) electrospun fiber mats were produced	The oil-containing fiber mat improved surface wettability by reducing the contact angle to approximately 30 ± 5°, enabling continuous and controlled release of the EO to enhance healing [52]
*Zataria multiflora*	A wound dressing was created by adding *Zataria multiflora* EO to poly(vinyl alcohol)-based nanofiber mats	The fiber mat with EO decreased the contact angle to around 30 ± 5°, improving surface wettability and providing sustained, controlled release to support tissue regeneration [53]
Eucalyptus	Hydrogels containing carboxymethyl chitosan (CMC) and carbomer 940 (CBM) were produced with EOs to create wound dressings	Eucalyptus oil-containing samples accelerated burn wound healing, achieving significant closure within approximately 48 h [54]
Ginger		
Cumin		
Wormwood	Wormwood EO (WEO) was encapsulated through an O/W Pickering emulsion method during the polymerization of GelMA, AM, and AAc-NHS to create a multifunctional hydrogel dressing (HD-WEO)	The hydrogel adhered effectively to skin tissue, maintaining EO stability and promoting healing of infected diabetic wounds [55]
*Melissa officinali L*	*Melissa officinalis L.* and *Anethum graveolens L.* EO were encapsulated into collagen hydrolysates extracted from bovine tendons and rabbit skins, blended with chitosan (CS), using the coaxial electrospinning technique for potential wound dressing applications	Synergistic stabilization of EOs within wound dressing matrices enhanced their therapeutic efficacy, resulting in improved wound healing outcomes [56]
*Anethum graveolens L*		
Oregano	PLCL/Silk fibroin nanofiber membranes were encapsulated with oregano oil to create a wound dressing	The wound dressing accelerated wound contraction and improved overall healing quality through the bioactive properties of oregano oil [57]
Oregano oil	A wound dressing containing zinc oxide coated with EOs, sodium alginate, hyaluronic acid, and silk fibroin was produced	Rapid wound healing was observed without infection risk; coating with ZnO and EO synergistically enhanced dressing functionality and degradation rate compared to clove oil [58]
Clove oil		
Mandarin	Antimicrobial wound dressings coated with silver nanoparticles, sodium alginate, and EOs were developed	Inclusion of volatile oils and silver nanoparticles enhanced the wound dressing’s antimicrobial effectiveness, promoting efficient and infection-free healing [59]
Clove Oil		
Niaouli		

In wound dressing and coating platforms, EOs have the potential for modulation of platform-wound interactions (Table 3). In a study by Üstündağ and Pişkin, *Calophyllum inophyllum* EO loaded fiber mats were created. It was observed that the oil containing fiber mat improved surface wettability by decreasing the contact angle to approximately 30 ± 5 (°) compared to the oil-free fiber mat [52]. In another study, it was found that Lavender oil increased the hydrophilicity of the wound dressing and contributed to wound healing when polyurethane nanofibers were loaded with lavender oil and silver nanoparticles [9]. Moreover, the coating platform created by encapsulating oregano oil into a nanofiber membrane has been reported to accelerate wound contraction and improve the quality of wound healing [57]. In another study, it was stated that the wound dressing formed by encapsulating wormwood EO in hydrogel provided the hydrogel to adhere to the skin tissue and thus contributed to the healing of infected diabetic wounds [55].

Recent studies are mostly attempts to integrate EOs with platforms to enrich their potential effects, but the findings in these studies have shown that the incorporation of EOs in wound dressing and coating platforms can have a wide range of effects. In fact, it has been reported that it may even contribute to the development of characteristics such as cooling effect, wettability, hydrophobicity, etc. that can modulate interactions at the platform: wound interface. However, the extensive study of the effects of these characteristics of these advanced platforms on living tissue is clearly needed.

## Challenges in the Application of Essential Oils

EOs offer many benefits in skin tissue engineering but face challenges such as instability to environmental factors, compatibility issues with biomaterials, and potential irritation or toxicity. Their complex production and variable yield further complicated use, necessitating ongoing research for safer, more effective applications.

### Stability and Volatility

The clinical use of EOs is limited by their volatility and instability, which compromise efficacy and safety [60]. EOs are complex blends of volatile compounds like terpenes and phenolics that easily evaporate or degrade when exposed to heat, light, humidity, or oxygen. This leads to loss of active ingredients and reduced therapeutic potential. Oxidative degradation further alters their chemical structure, diminishing bioactivity and affecting physical properties like color, viscosity, and odor [14].

To address these issues, production and storage conditions must be optimized. Advanced formulations-such as microencapsulation, nanoemulsions, liposomes, and polymer-based matrices-help protect EOs from degradation and enable sustained release [60]. These systems are especially valuable in skin tissue engineering for localized delivery and reduced toxicity. Adding antioxidants and UV blockers, along with controlled processing environments, further enhances EO stability [12]. Overcoming EO volatility and instability requires a multidisciplinary approach. Combining chemistry, materials science, and biomedical engineering can enable the development of effective EO-based wound dressings and scaffolds for regenerative medicine.

### Interactions with Biomaterials

EOs are used in skin tissue engineering with various materials or carrier systems because they have negative results such as stability, volatility and safety when used in pure form. The interaction of these oils with biomaterials can cause physical and chemical changes in the material. Especially in polymer-based systems, it can negatively change the crystallization structure, mechanical properties of the polymer such as viscosity, flexibility or shape, and thermal properties. These changes in the material’s characteristics can cause challenges to the end-use of the material, leading to a decrease in the anticipated impact [58]. For example, as a result of the integration of *Tanacetum polycephalus* oil into wound dressing platforms, it has been observed that the hydrophilicity of the structure decreases and the degree of swelling decreases due to the hydrophobicity of the EO, which may provide an opportunity or potential drawback depending on the type of wound [20]. In another study, it was observed that when coriander EO was added to the film, the strength and tensile properties of the film decreased [11]. In the incorporation of EOs into biomaterial platforms, the EO to be integrated should be properly screened and the platform design and manufacturing strategy should be optimized to avoid any loss of platform characteristics.

### Formulation Challenges

EOs provide numerous benefits in skin tissue engineering; however, formulation challenges persist, particularly regarding the determination of their optimal concentration. Due to the presence of potent bioactive compounds, high concentrations of EOs can lead to adverse effects such as skin irritation, inflammation, redness, allergic reactions, and toxicity. Conversely, concentrations that are too low may result in insufficient therapeutic efficacy. Therefore, careful optimization of EO dosage is critical to balance maximizing their beneficial effects while minimizing potential cytotoxicity and irritation in skin applications. For example, it has been stated that lemongrass EO showed 80% cell viability when used in low concentrations, while this viability decreased to 35% when used in high concentrations, showing a cytotoxic effect [13]. In addition, a study conducted by Miranda et al. emphasized the importance of using EOs in appropriate doses, as the use of tea tree oil in high concentrations caused IL-8 suppression in fibroblast cells and showed a toxic effect [29]. In skin tissue engineering, accurately determining the EO dose is crucial for safe and effective use due to direct skin contact. Homogeneous distribution of EOs in formulations is challenging because their lipophilic nature hinders even dispersion in water-based biomaterials. This uneven distribution leads to inconsistent biological activity at the treatment site and causes rapid release, limiting long-term efficacy. Therefore, incorporating EOs into suitable carrier or nanotechnology-based delivery systems is essential to ensure controlled, sustained therapeutic effects.

Variations in plant species, growth conditions, and plant parts used lead to significant differences in EO composition. Factors such as soil, climate, temperature, and humidity affect the oil’s chemical profile, even among species of the same plant. Consequently, EOs from distinct sources exhibit varying effectiveness and stability-for instance, one species’ oil may have antimicrobial properties while another’s may be primarily anti-inflammatory. Additionally, the same oil may affect pathogens or cell types differently, producing either beneficial or adverse effects depending on its specific composition. For instance, it has been reported that the antioxidant properties of *Mentha piperita* EO were lower than those reported in the literature. Although the plant used in the study was the same as the plant mentioned in the literature, it was stated that this effect was due to differences between the species [34]. While an EO provides advantages by supporting fibroblast cell proliferation, it can also support the proliferation of cancer cells, which can lead to undesired complications. Therefore, prior to the application of EOs in skin tissue engineering, it is imperative to thoroughly characterize their extraction methods, elucidate their molecular targets and affected cellular pathways, and comprehensively evaluate their pharmacological properties and bioactivities.

### Scalability and Cost-Effectiveness

In tissue engineering, scalability and cost-effectiveness are crucial for the development of EOs for translational medicine and practical applications. Although the superior properties of EOs supported by current studies offer them as unique tools in medical applications, shortcomings in product development are noteworthy due to compositional and efficacy concerns [61].

Particularly, the variation of EO components depending on the plant species, geographical origin and extraction methods hinders the optimization and standardization processes to be implemented during their product development. Furthermore, the extraction and purification processes of EOs require large amounts of plant material and energy, which is a significant limitation in terms of production costs and sustainability [62]. Cost concerns are not limited to these. EO-integrated tissue engineering applications involve advanced technologies, complex manufacturing processes and additional costs [63]. Another cost factor is the balance between EO concentration and potential cytotoxicity. Such an optimization process can be achieved through precise formulation and quality assurance control mechanisms.

In conclusion, while EOs have significant therapeutic potential in tissue engineering, their scalable and cost-effective production requires optimization of EO extraction, standardization and formulation processes. While advances in nanotechnology and polymer science have enabled more efficient integration of EOs, further research is still required to simplify production and reduce costs for widespread clinical and commercial use.

## Current Evidence and Future Clinical Directions

The integration of essential oils into biomaterial-based structures demonstrates multifunctional therapeutic effects in skin tissue engineering. Preclinical studies show that EO-functionalized matrices exhibit significant antimicrobial activity against common wound pathogens such as *Staphylococcus aureus, Staphylococcus epidermidis, and Candida albicans*. Beyond infection control, EOs have been reported to modulate inflammatory responses and enhance fibroblast proliferation and migration. For example, a recent study demonstrated that multilayered PVA/gelatin nanofibers containing *Tanacetum polycephalum* essential oil, or used in combination with amoxicillin, promoted cytocompatibility and fibroblast adhesion. Furthermore, they showed a 99.99% reduction in bacteria against both Gram-positive and Gram-negative strains. EO-containing structures have also been reported to enhance fibroblast migration in scratch tests and accelerate wound closure in full-thickness animal models. Histological analyses also revealed increased angiogenesis, enhanced collagen deposition, improved re-epithelialization, and reduced inflammation compared to controls. These results highlight the antimicrobial and regenerative potential of EO-functionalized structures [20]. In another study, topical formulations of nanostructured lipid carriers containing rosemary essential oil derived from *Rosmarinus officinalis* demonstrated significant antibacterial activity against pathogens, including *Staphylococcus aureus *and* Pseudomonas aeruginosa*, in a mouse model of infected full-thickness wounds. Compared to untreated controls, wounds treated with this structure showed reduced bacterial colonization, accelerated wound contraction, and earlier epithelialization (day 12 compared to day 16 in controls). Increased fibroblast infiltration, increased collagen deposition, and improved neovascularization were also reported. These regenerative effects were accompanied by increased systemic IL-3, IL-10, VEGF, and SDF-1α levels. These results suggest that advanced systems can enhance the stability, bioavailability, and therapeutic efficacy of essential oils in infected wound environments [64]. A study by El Banna et al. investigated the wound healing efficacy of a multifunctional topical formulation in an excision wound model in rats. The formulation (Grotto cream) contained beeswax, d-panthenol, lavender essential oil, glycerin, vitamin E, allantoin, and dimethicone. This formulation was compared to a commercially available antibiotic cream (Fucidin) containing 2% fusidic acid and a cream base used as a control. Both normal and streptozotocin-induced diabetic rats were divided into three parallel groups: control, Grotto cream-treated, and antibiotic-treated (n = 10 in each group). Standardized circular excision wounds were created, and topical treatments were applied every three days. Normal animals were monitored for 21 days after wound creation, while diabetic animals, representing an impaired healing pattern, were followed for 30 days. The results indicated that Grotto cream application accelerated wound healing compared to the control group. In both normal and diabetic rats, the wound contraction rate was significantly increased and the epithelialization time was shortened. Furthermore, Grotto cream was found to be more effective than the reference drug, Fucidin cream [65]. In addition to biomaterial-based *in vivo* models, mechanistic in vitro studies using human dermal fibroblasts provide further insight into the biological activity of essential oils (EOs) on skin cells. In a pre-inflamed human dermal fibroblast system mimicking chronic inflammatory states, various essential oils have been shown to significantly modulate biomarkers associated with inflammation, immune regulation, and tissue remodeling. Specifically, bergamot oil from *Citrus bergamia,* coriander oil from *Coriandrum sativum*, and spikenard oil from *Nardostachys jatamansi* have been reported to significantly inhibit pro-inflammatory mediators such as MCP-1, VCAM-1, and ICAM-1, as well as proteins involved in tissue remodeling, including collagen I/III and TIMPs. Other oils, such as *Helichrysum italicum* and *Cananga odorata*, have also been shown to modulate tissue remodeling pathways [66].

Despite these promising preclinical findings, clinical trials of EO-loaded tissue-engineered constructs remain limited. Much of the current data highlights the clinical safety and translational potential of advanced constructs and topical EO-based formulations. Better-designed clinical trials are needed to validate efficacy and long-term safety in human wound healing applications.

## Future Perspectives

Promising results have been demonstrated in various studies on the use of EOs in skin tissue engineering; however, significant challenges and research gaps remain. A major limitation is the lack of *in vivo* and clinical evaluations, which are essential to confirm the therapeutic efficacy, safety, and long-term biocompatibility of EO-based formulations. Future research should focus on comprehensive animal studies and controlled clinical trials to ensure that laboratory findings translate effectively into real-world applications.

Optimization of carrier systems such as hydrogels, nanoparticles, and electrospun fibers is also an important issue to address. Improving the mechanical properties, homogeneity, and release kinetics of these systems is important for better adaptation to the physiological requirements of skin tissue regeneration. Technologies such as wet spinning and nanocarrier loading in particular need to be developed to provide consistency and reproducibility.

The molecular mechanisms of many EO components, including cuminic aldehyde, neomenthol, and carvacrol, remain insufficiently understood. Investigating their antibacterial, anti-inflammatory, and anticancer pathways is vital for designing targeted therapies. Although essential oils have demonstrated anticancer activity in various tumor models through mechanisms such as apoptosis induction, oxidative stress modulation, and cell cycle arrest, reports specifically addressing skin cancer applications remain very limited. Considering the increasing incidence of melanoma and non-melanoma skin cancers, the integration of EO-based delivery systems into skin-targeted therapeutic platforms represents a promising yet underexplored research direction. Future studies should focus on skin-specific models, localized delivery strategies, safety profiling, and translational validation to determine the feasibility of EO-based systems in skin cancer treatment. Expanding studies across diverse cell lines and microbial species will increase the generalizability of the results. Exploring synergistic combinations of EOs with existing antimicrobial agents or growth factors presents another promising strategy, potentially enhancing treatment outcomes for chronic wounds and skin cancer prevention. Moreover, clinical evaluations involving volunteer groups are necessary to assess patient-centered outcomes such as tolerability and formulation preferences, guiding the development of more effective and user-friendly EO-based therapies.

## Conclusion

Essential oils, which are naturally sourced and biocompatible, stand out as promising bioactive agents in skin tissue engineering, especially in wound healing and skin regeneration applications, thanks to their properties such as antimicrobial, anticancer and supporting cell renewal. However, the usage limitations of essential oils, such as formulation stability, dosage control and potential cytotoxicity, are overcome by providing effective and controlled release through their use with carrier systems. Although current studies support the *in vitro* and *in vivo* efficacy of these oils, more comprehensive and standardized research is required for transition to clinical applications. Future studies should focus on elucidating the mechanisms of action of essential oils at the cellular level and clarifying the safe usage limits.

## Data Availability

Data supporting the findings of this study are available from the corresponding author upon reasonable request.
